# Targeting T-Cell Activation for Malaria Immunotherapy: Scoping Review

**DOI:** 10.3390/pathogens14010071

**Published:** 2025-01-14

**Authors:** Balsa Nobility Gustifante, Shafia Khairani, Nisa Fauziah, Silvita Fitri Riswari, Afiat Berbudi

**Affiliations:** 1 Medical Undergraduate Study Program, Faculty of Medicine, Universitas Padjadjaran, Bandung 45363, Indonesia; balsa21001@mail.unpad.ac.id; 2 Veterinary Medicine Program, Faculty of Medicine, Universitas Padjadjaran, Bandung 45363, Indonesia; shafia@unpad.ac.id; 3 Department of Biomedical Sciences, Cell Biology Division, Faculty of Medicine, Universitas Padjadjaran, Bandung 45363, Indonesia; 4 Department of Biomedical Sciences, Parasitology Division, Faculty of Medicine, Universitas Padjadjaran, Bandung 45363, Indonesia; nisa@unpad.ac.id (N.F.); silvita.fitri@unpad.ac.id (S.F.R.)

**Keywords:** T-cell, cell-mediated immunity, immunotherapy, malaria, *Plasmodium*

## Abstract

Malaria remains a critical global health issue due to high mortality rates, drug resistance, and low treatment efficacy. The genetic variability of *Plasmodium* proteins complicates the development of long-lasting immunity, as it impedes the human immune system’s ability to sustain effective responses. T cells play a crucial role in combating malaria, but the parasite’s complex life cycle—spanning liver and blood stages—presents significant challenges in effectively activating and targeting these cells. Immunotherapy, which enhances the immune response and promotes durable T cell activity, offers a promising avenue for more effective and lasting malaria treatments. This review systematically analyzed 63 studies published in the last decade, focusing on the role of T cells in malaria. Among the studies, 87.2% targeted T cells as immunotherapy candidates, with CD4+ and CD8+ T cells each accounting for 47.6% of the studies. γδ T cells were the focus in 7.9% of cases, while 12.7% explored non-T cell contributions to enhancing T cell-mediated responses. The findings underscore the potential of T cells, particularly CD8+ T cells, in liver-stage defense and advocate for the exploration of advanced vaccine platforms and novel therapies, such as mRNA-based vectors and monoclonal antibodies.

## Simple Summary

Efforts to control malaria, a devastating parasitic disease, are continually challenged by its high mortality rates, the emergence of drug-resistant strains, and the limited durability of current treatments and vaccines. This review highlights the critical role of T cells in malaria immunity, with a particular focus on CD8+ T cells during the liver stage of infection. These cells are essential in combating malaria but face challenges due to the parasite’s complex life cycle and immune evasion strategies. The review underscores the promise of immunotherapy, including mRNA-based vaccines and monoclonal antibodies, in enhancing T-cell activity and achieving durable immune responses. While advances like the RTS,S and R21 vaccines mark progress, their limited efficacy and reliance on booster doses highlight the need for more effective approaches. Future research should prioritize optimizing T-cell-targeted therapies, addressing existing knowledge gaps, and exploring innovative immunotherapy platforms to advance malaria control and eradication efforts.

## 1. Introduction

Malaria, a parasitic disease caused by *Plasmodium* species, continues to be a major global health challenge, disproportionately affecting subtropical and tropical regions, particularly in Africa. In 2022, the disease was responsible for 619,000 deaths, an increase from 568,000 in 2019, with 247 million cases reported globally [1]. The migration of travelers from endemic areas further facilitates its spread to non-endemic regions, exacerbating the global burden of the disease [2]. In endemic areas, malaria is a leading cause of morbidity and mortality, imposing a significant health and economic burden on affected nations.

Although malaria is curable with prompt treatment, complications such as cerebral malaria, coma, and death can arise if left untreated. The *Plasmodium* parasite’s complex, multi-stage life cycle—spanning liver and blood stages—requires a coordinated host immune response. *Plasmodium falciparum* is responsible for most severe cases, while species like *Plasmodium vivax* and *Plasmodium ovale* can form dormant stages that reactivate, causing relapses [3].

Advancements in antimalarial treatment have evolved from monotherapy, targeting the parasite with a single drug, to combination therapies that employ multiple mechanisms. Despite these developments, the emergence of drug-resistant strains has significantly diminished treatment efficacy [4,5]. Vaccines, one of the oldest forms of immunotherapy, have also been pursued as a long-term solution. To date, the World Health Organization (WHO) has approved two malaria vaccines: RTS,S/AS01 (2021) and R21/Matrix-M (2023). These vaccines target specific *Plasmodium* epitopes to trigger adaptive immune responses and establish memory. However, the parasite’s extensive protein variability complicates target selection and limits vaccine efficacy [6,7].

Clinical trials have revealed that the RTS,S vaccine provides only 55.8% median efficacy in African children, with a rapid decline in immune protection within six months. In contrast, the R21 vaccine demonstrates a higher efficacy of 77% and a more sustained immune response but still requires booster doses [8]. The search for more effective, long-lasting solutions remains imperative.

**Figure 1:**
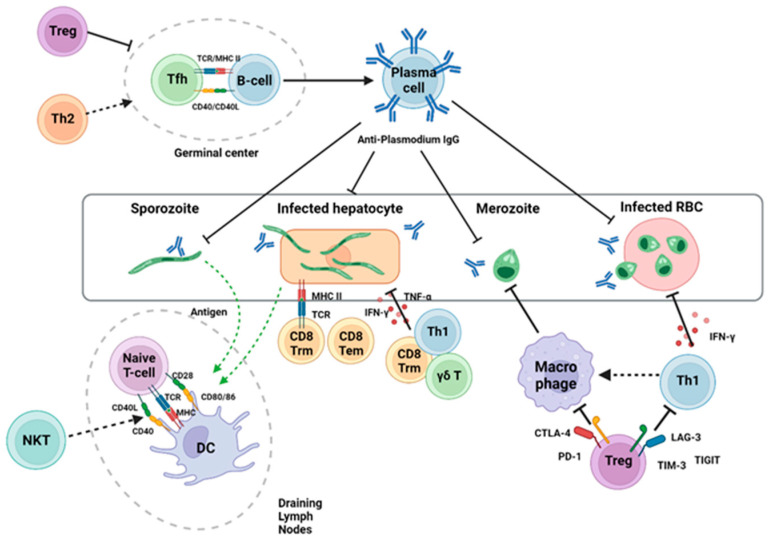
T-cell role in malaria infection. This figure depicts the immune response against *Plasmodium* infection, highlighting the roles of both innate and adaptive immunity, with a focus on T cell activation. Innate immune cells, such as dendritic cells (APCs), are essential for the initial detection of *Plasmodium* and for initiating adaptive immune responses. T cells, upon activation in the lymph node, contribute to both cellular and humoral immunity. Cellular immunity is primarily mediated by CD8+ cytotoxic T cells and Th1 cells, which target and eliminate infected cells. On the other hand, humoral immunity is facilitated by the activation of B cells through T follicular helper (Tfh) cells, leading to the production of antibodies that provide protection against *Plasmodium* infection. This integrated immune response is crucial for controlling the parasite and limiting the progression of malaria.

T cells, key mediators of adaptive immunity, play a central role in combating malaria (Figure 1). CD4+ and CD8+ T cells contribute to immune responses at different stages of infection, while regulatory T cells (Tregs) modulate these responses. However, the complexity of immune evasion strategies employed by *Plasmodium* necessitates further research into enhancing T-cell activation and sustaining their efficacy [9,10]. Recent advancements in immunotherapy, including mRNA-based vaccine platforms and monoclonal antibodies, offer promising avenues for addressing these challenges.

This review explores the potential of T cells as critical targets for malaria immunotherapy, emphasizing their diverse roles and mechanisms of action. Understanding these pathways could pave the way for innovative therapeutic strategies to advance global malaria control and eradication efforts.

## 2. Materials and Methods

This scoping review was conducted following the Joanna Briggs Institute (JBI) Manual for Evidence Synthesis, with detailed procedural guidance provided by the Arksey and O’Malley framework. The review was reported in accordance with the PRISMA-ScR (Preferred Reporting Items for Systematic Reviews and Meta-Analyses extension for Scoping Reviews) guidelines [11]. The protocol for this scoping review has been registered in the OSF Registries database and is publicly accessible at https://doi.org/10.17605/OSF.IO/47W9X (accessed on 27 December 2024). A systematic search strategy was developed, combining specific keywords with Boolean operators, and applied across four online databases: PubMed, Scopus, Cochrane Library, and Google Scholar. In addition, a snowball search was performed to identify the potentially relevant literature not captured during the initial database search. The titles and abstracts of identified studies were screened to exclude irrelevant articles, followed by a full-text review conducted and independently (blinded) by two reviewers. The first reviewer extracted data focusing on the role of T cells in malaria immunotherapy, stages of immune response, specific immune mechanisms, study type, and results.

### 2.1. Eligibility Criteria

The review included original research articles published between 29 May 2014 and 29 May 2024. Both experimental and non-experimental studies were considered, encompassing in vivo and in vitro research, provided they were published in English and available in full-text. Articles excluded from the review included commentaries, editorials, and review papers. References cited in eligible articles were further examined to identify additional relevant studies, ensuring coverage within the inclusion time frame. Articles deemed irrelevant to the scope of this review were excluded.

### 2.2. Search and Screening Process

The literature search identified 104 articles. Following title and abstract screening, 37 articles were excluded for reasons such as lack of original research (26 articles), non-specific focus on malaria (6 articles), or irrelevance to immunotherapy (5 articles). Full-text screening of 67 articles resulted in the exclusion of 4 articles, leaving 63 studies in the final review.

### 2.3. Data Extraction and Analysis

The data extraction process emphasized identifying T-cell targets, immune response stages, mechanisms of action, study design, and experimental outcomes. Studies included research on various *Plasmodium* species, including *Plasmodium falciparum* and *Plasmodium* vivax in human subjects, as well as *Plasmodium berghei*, *Plasmodium yoelii*, and *Plasmodium chabaudi* in murine models. Some studies also involved immunized murine models with *P. falciparum* sporozoites to simulate immune responses relevant to human malaria.

The dual approach of advanced database searching and snowball sampling ensured comprehensive coverage of the relevant literature. A final synthesis integrated findings from all included studies, highlighting immunotherapy targets and mechanisms involving T cells in malaria.

## 3. Results

### 3.1. Literature Search Results

**Figure 2:**
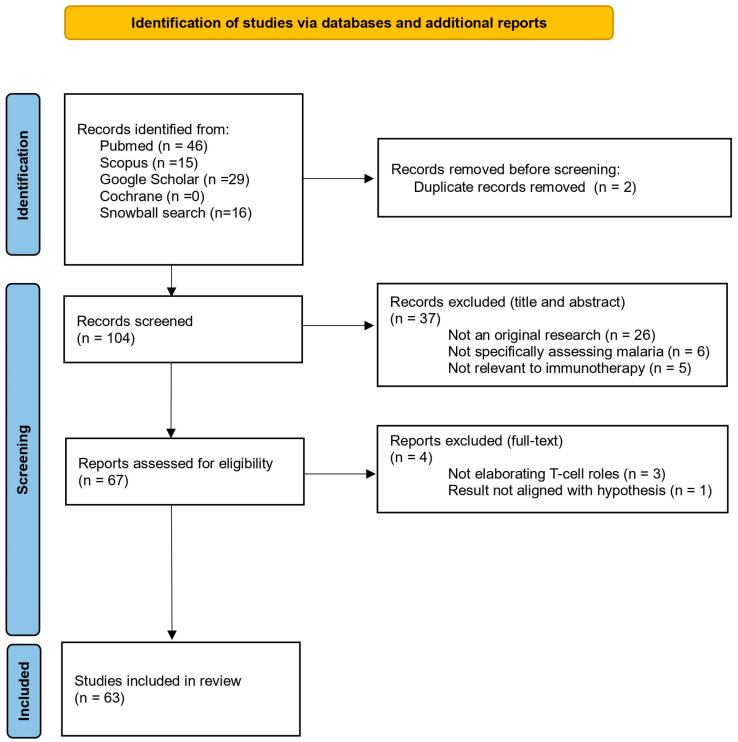
PRISMA-ScR flow diagram for included studies.

The literature search flowchart is shown in Figure 2. A total of 104 articles were initially identified and screened based on their titles and abstracts, resulting in 67 articles selected for full-text eligibility screening. A total of 63 literature works was obtained as the final inclusion.

In the context of malaria host immune response, 7 articles (11.1%) focused on T-cell targets during the pre-activation phase, 51 (81%) on the activation phase, and 5 (7.9%) on the post-activation phase. The roles and pathways of T-cell immunity are not mediated by T-cells alone but also involve interactions with other cells from the innate immune system. Thus, 87.2% (55/63) of the articles focused solely on T cells as immunotherapy targets, while the remaining 12.7% (8/63) examined the roles of non-T cells that complement the overall immune response.

Among the studies targeting T cells, 47.6% (30/63) focused on CD4+ T cells, 47.6% (30/63) on CD8+ T cells, 15.8% (10/63) on both CD4+ and CD8+ T cells, and 7.9% (5/63) on γδ T cells. Non-T cells that were empirically found to support T-cell function and are potential targets for malaria immunotherapy include dendritic cells (9.5%, 6/63), particularly CD8α+ DCs, macrophages, polymorphonuclear myeloid-derived suppressor cells (3.2%, 2/63), and natural killer T (NKT) cells (3.2%, 2/63).

### 3.2. Literature Search Overview

The systematic search initially identified 104 articles across four databases and additional sources through snowball sampling. After removing two duplicates, 102 unique records were screened by titles and abstracts, excluding 37 that did not meet the inclusion criteria: 26 were non-original research articles, 6 lacked a specific focus on malaria, and 5 were irrelevant to immunotherapy. The remaining 67 articles underwent full-text review, resulting in the exclusion of 4 studies for failing to elaborate on T-cell roles or align with the review’s scope. Ultimately, 63 studies were included in the final analysis. Figure 2 outlines the search and screening process.

### 3.3. Study Characteristics

The included studies encompassed both experimental and observational research on malaria immunotherapy, focusing on T cells across various stages of immune response. Research subjects included *Plasmodium*-infected or immunized human and murine models, with the majority assessing *Plasmodium falciparum* and *Plasmodium vivax* in humans and *P. berghei*, *P. yoelii*, and *P. chabaudi* in mice. These studies examined diverse T-cell-related mechanisms and therapeutic strategies, ranging from experimental vaccines to novel immunomodulatory approaches. The specific keywords and search strategies applied in the databases are summarized in Table 1, providing transparency about the methods used to capture relevant studies.

### 3.4. Focus of T-Cell Studies

Among the 63 included studies, 87.2% (55/63) specifically targeted T cells as immunotherapy candidates, while 12.7% (8/63) investigated non-T-cell components that enhance T-cell-mediated immunity. The majority of studies (81%) centered on T-cell activation during malaria infection, while 11.1% examined pre-activation processes, and 7.9% explored post-activation phases.

### 3.5. T-Cell Subtype Focus

T cells are critical players in the immune response to *Plasmodium* infection, with different subtypes contributing distinct roles at various stages of the disease. The literature reviewed reveals that multiple T-cell subsets are involved in the host defense against malaria, each with specific functions that help combat the parasite. In particular, CD4+ and CD8+ T cells are the most frequently studied for their roles in both cellular and humoral immunity. Additionally, less-explored subsets such as γδ T cells have also been identified as important contributors to innate-like immunity and early defense mechanisms. The following provides a summary of the focus on key T-cell subtypes in the literature:

- CD4+ T cells: Addressed in 47.6% (30/63) of studies, these cells play a key role in orchestrating immune responses, particularly in the erythrocytic stage of malaria.
- CD8+ T cells: Also highlighted in 47.6% (30/63) of studies, with a predominant focus on their role in liver-stage immunity.
- γδ T cells: Investigated in 7.9% (5/63) of studies for their unique contributions to innate-like immunity and interactions with other immune components.

### 3.6. Non-T-Cell Contributions

Studies addressing non-T-cell immune components emphasized their supportive roles in T-cell activation and function. For instance, 9.5% (6/63) focused on dendritic cells, which enhance antigen presentation to T cells, while macrophages and polymorphonuclear myeloid-derived suppressor cells (3.2% each) were also studied for their roles in modulating T-cell responses.

### 3.7. Integrated Findings and Implications

The studies reviewed demonstrate the multifaceted roles of T cells in malaria immunotherapy. Most notably, T-cell activation emerged as a central process for effective malaria defense, with CD8+ T cells being crucial in liver-stage immunity and memory T-cell formation, ensuring long-term protection. The analysis also highlighted γδ T cells and non-T-cell components, such as dendritic cells and macrophages, as integral parts of the broader immune landscape.

In addition, the immunotherapy targets identified are summarized in Table 2, which categorizes them based on T-cell activation stages (pre-activation, activation, and post-activation), highlighting their mechanisms of action and experimental outcomes. For instance, CD8+ T cells demonstrated significant potential in liver-stage immunity, supported by pre-activation enhancements through dendritic cells.

### 3.8. Interpretation of Results

These findings underscore the importance of tailoring immunotherapeutic strategies to enhance T-cell functionality and longevity while incorporating supportive roles of non-T-cell components. Such a multifaceted approach may provide a more robust defense against malaria and pave the way for innovative treatments.

## 4. Discussion

### 4.1. T-Cell Activation and Mechanisms

T cells progress through distinct stages in their immunological response to malaria before fully developing the ability to combat *Plasmodium*. These stages can be categorized into three phases: pre-activation, activation, and post-activation. Each phase involves critical interactions with antigen-presenting cells (APCs) and regulatory molecules that shape the immune response.

This figure illustrates the pre-activation phase of T cell responses during *Plasmodium* infection. It depicts the recognition of *Plasmodium* antigens by innate immune cells, including dendritic cells, macrophages, and other antigen-presenting cells (APCs). These cells play a crucial role in detecting the parasite and initiating the immune response. Upon encountering *Plasmodium*, the APCs process and present the antigens to naïve T cells, triggering their activation. This process is essential for the subsequent adaptive immune response, including the activation of CD4+ and CD8+ T cells that contribute to the defense against malaria.

### 4.2. Pre-Activation Phase

**Figure 3:**
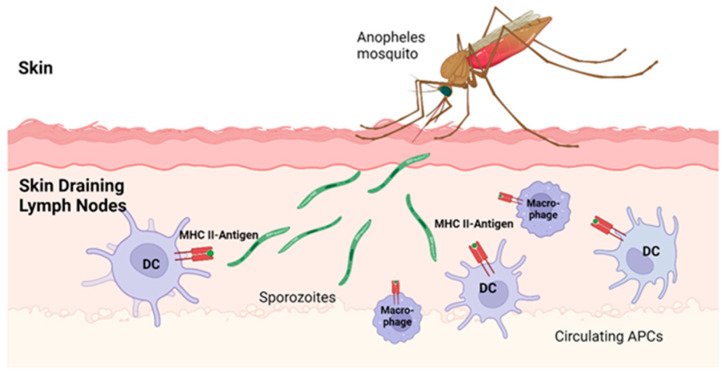
T-Cell pre-activation stage.

The pre-activation phase begins when *Plasmodium* sporozoites invade the host and travel to the skin-draining lymph nodes (dLNs). Circulating dendritic cells (DCs) and macrophages, functioning as APCs, patrol these areas, recognize *Plasmodium* antigens, and transport them for presentation to T cells [73,74]. Studies by Daniel et al. and Tan et al. highlight the potential of targeting toll-like receptors (TLRs) on DCs and macrophages to enhance their activation, resulting in heightened responsiveness to *Plasmodium* antigens and improved sensitivity to sporozoites [12,13] (Figure 3).

Within this phase, specific subsets of DCs, such as CD8α+ DCs, have been identified as particularly efficient in antigen presentation. However, their activity can be modulated or suppressed by γδ T cells, underscoring the complex interplay of immune components in shaping the host response to malaria [15,16].

### 4.3. Activation Phase

**Figure 4:**
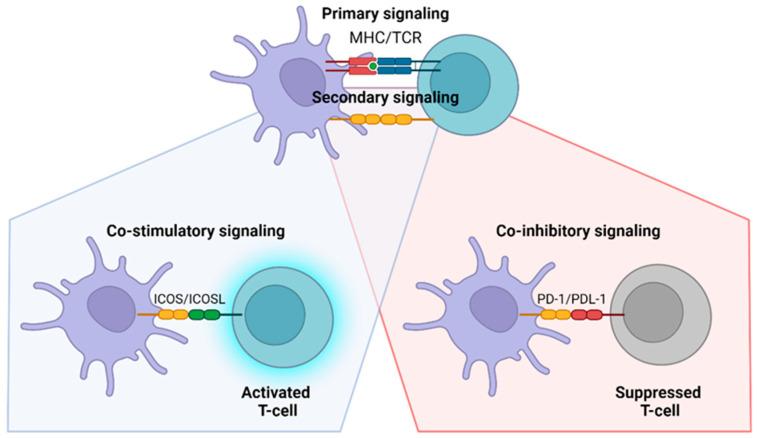
T-Cell Activation Stage.

T-cell priming and activation occur during this phase. This process is initiated when the T-cell receptor (TCR) binds to the major histocompatibility complex (MHC) on APCs, coupled with TLR engagement. These interactions trigger T-cell activation, enabling them to perform key functions such as eliminating infected cells and coordinating broader immune responses (Figure 4).

Studies have elucidated the specific roles of T-cell subsets during malaria infection. CD8+ T cells are essential for liver-stage immunity (pre-erythrocytic phase), while CD4+ T cells dominate during the erythrocytic stage by supporting cytokine production and antibody responses [20,21,36,37,38,39,40,41]. For instance, Pichugin et al. demonstrated that the binding of MHC I to CD8+ T cells is reliant on a transporter-associated antigen processing (TAP)-dependent pathway, emphasizing the importance of antigen presentation mechanisms [24].

Receptor engagement also plays a pivotal role in T-cell activation. Stimulatory receptors such as ICOS and OX40 amplify immune responses, while inhibitory receptors, including PD-1, CTLA-4, TIM-3, LAG-3, and TIGIT, suppress T-cell function to regulate immune activation and prevent overreaction [44,50,51,52,58,59]. Additionally, the IL-27R receptor has been identified as a critical regulator of IL-10 production, further modulating T-cell responses during malaria infection [57].

This figure illustrates the activation of T cells in the lymph node during *Plasmodium* infection. Dendritic cells (DCs) present *Plasmodium* antigens to T cell receptors (TCR) on naïve T cells, initiating T cell activation. Successful activation requires not only the primary MHC/TCR interaction but also co-stimulation through secondary signaling, specifically the binding of B7 proteins on APCs with CD28 on T cells. This co-stimulation is crucial for full T cell activation and proliferation. Additionally, the figure highlights the role of immune checkpoint molecules, such as PD-1 on T cells and PD-L1 on APCs, which mediate co-stimulatory inhibition. When PD-1/PD-L1 interactions occur, T cell activation is suppressed, leading to an ineffective immune response and potentially contributing to immune evasion by *Plasmodium*. This process underscores the importance of both positive and negative regulatory signals in shaping the immune response against malaria.

### 4.4. Post-Activation Phase

In the final phase of T-cell activation, effector T cells (Teff) perform cytotoxic functions and secrete cytokines to combat *Plasmodium*, reducing parasitemia until the contraction phase begins. During this period, some Teff cells transition into memory T cells (Tmem), which are crucial for long-term immunity and protection against reinfections [67,68,69].

Recent research by Ibitokou et al. identified the fatty acid synthesis (FAS) pathway as a critical mechanism for generating Tmem cells, underscoring the metabolic requirements for sustained immune memory [68]. Additionally, innate immune cells provide significant support during this phase by enhancing T-cell function and promoting adaptive responses, further strengthening the host’s defense mechanisms [71].

By understanding these distinct phases of T-cell activation and their regulatory mechanisms, targeted immunotherapeutic strategies can be developed to enhance T-cell efficacy and longevity, providing a robust defense against malaria.

### 4.5. CD8+ T Cells as Immunotherapy Targets in Malaria

CD8+ T cells differentiate into several subsets with specialized roles in immunity. These include memory precursor effector cells (MPEC), which further divide into central memory T cells (TCM), tissue-resident memory T cells (TRM), and effector memory T cells (TEM). Short-lived effector cells (SLECs), composed of cytotoxic T lymphocytes (CTLs), execute immediate responses, while exhausted T cells (TEX) represent a subset with diminished functionality [75].

In malaria, parasite-specific CD8+ T cells are pivotal for defending against both liver-stage and blood-stage infections [76]. Most studies in this review emphasize CD8+ T-cell priming during the liver stage, supported by 14 studies [19,20,21,22,23,24,25,26,27,28,29,30,31,32]. This focus arises from the importance of early immune intervention in preventing disease progression before the parasite reaches the erythrocytic stage. Targeting the relatively low number of sporozoites infecting hepatocytes offers an effective strategy for immunity, as the parasite burden increases significantly once it enters the bloodstream [76].

In addition to their cytotoxic functions, liver-resident TRM cells have drawn substantial research attention due to their potential for providing durable protection against reinfections [31]. Experiments tracking TRM-like cells, identified by the common TRM receptor CD69, revealed a similar protective role during the liver stage [33]. These findings have inspired the development of prime-and-trap vaccines designed to prime CD8+ T cells and retain them in the liver to differentiate into TRM cells. 

Fernandez-Ruiz et al. further confirmed the unique patrolling qualities of liver-resident TRM cells, which distinguish them from TEM cells [22]. This highlights their critical role in maintaining liver-stage immunity. These insights address prior evidence suggesting that while blood-stage defenses are effective, immunity against liver-stage malaria has often been insufficient. Enhancing liver-stage immunity through CD8+ T-cell-focused interventions represents a promising avenue for advancing malaria immunotherapy.

### 4.6. Targeting CD4+ T-Cell in Malaria Immunotherapy

Naïve CD4+ T cells possess the ability to differentiate into multiple lineages, including Th1, Th2, Th17, T follicular helper (Tfh), and regulatory T cells (Treg), depending on the cytokine environment and signaling pathways. These lineages play distinct roles in adaptive immunity, with CD4+ T cells being central to protective responses during the erythrocytic stage of malaria. They prime macrophages for phagocytosis and signal B cells to produce antibodies against *Plasmodium* [77].

Th1 cells contribute to early protection by producing pro-inflammatory cytokines that target intracellular *Plasmodium*, while Th2 cells mediate B cell activation, supporting the production of antibodies for extracellular defense [78]. Recent research has underscored the critical role of Tfh cells in T-cell-dependent antibody production, as they induce B cell differentiation in germinal centers (GC), forming a vital link to humoral immunity [44,45,46,47,48,49]. During malaria infection, a balance of Th1/Tfh is required to effectively control the parasite [43]. Mechanisms identified by Jian et al. highlighted the importance of CD40 signaling, while Salles et al. identified P2X7 expression, both of which direct differentiation into Th1 cells and help regulate Tfh cell numbers to maintain a balanced immune response [42,43].

Several studies have reported that inhibitory molecules downregulate IFN-γ-producing Th1 activity, thereby limiting the immune response to *Plasmodium* and underscoring the importance of Th1 cell magnitude in malaria protection [53,59]. The antagonistic relationship between Th1 and Th2 [78] cells is further supported by a study conducted by Coomes et al., which observed that in *Plasmodium* and Helminth co-infection, a higher proportion of functional Th2 cells was present. As a result, a robust Th2 response provides extracellular protection against Helminth but diminishes control over *Plasmodium* [64]. Th1 cells are considered superior in malaria protection due to their intracellular mechanisms of *Plasmodium*. Another lineage, Th17, works in tandem with Treg cells to modulate protective immunity. Imbalances in Th17 and Treg populations can be predicted and tracked by monitoring associated cytokines such as TGF-β and IL-6, which regulate their differentiation [62,63].

The diverse functionalities of CD4+ T cells extend beyond the erythrocytic stage. In a seminal study, Tsuji (1990) reported the characterization of a CD4+ T cell clone (A1.6) derived from mice immunized with irradiated sporozoites. This clone, which recognizes a plasmodial antigen distinct from the circumsporozoite protein, exhibited cytotoxic activity and produced IFN-γ and IL-2 in vitro. Remarkably, passive transfer of this clone into naïve mice conferred significant protection against sporozoite challenge, demonstrating its potential in immunotherapeutic strategies [79]. This finding underscores the versatility of CD4+ T cells and their profound implications for advancing malaria immunotherapy.

Ultimately, the multifaceted roles of CD4+ T cells—ranging from intracellular parasite control by Th1 cells to antibody production mediated by Tfh cells—highlight their indispensability in malaria immunity. The integration of historical insights, such as those provided by Tsuji (1990), with contemporary studies illuminates the pathway for designing targeted immunotherapeutic strategies to combat malaria effectively.

### 4.7. γδ T-Cell Findings

γδ T-cells, which account for approximately 4% of all T-cells in adult humans, are derived from a distinct progenitor that is neither CD4+ nor CD8+. These cells express a unique γδ T-cell receptor (TCR) that distinguishes them from conventional αβ T-cells. Although the functional roles of γδ T-cells in infectious diseases remain incompletely understood [76], early studies began unraveling their significance in the context of malaria immunity. For instance, Tsuji et al. (1994) demonstrated that γδ T-cells play a critical role in protective immunity against malaria, particularly during the liver stage. Their study using *Plasmodium yoelii*, a rodent malaria model, revealed that the depletion of γδ T-cells in αβ T-cell-deficient mice abrogated the protective immune response induced by immunization with irradiated sporozoites. Furthermore, the adoptive transfer of γδ T-cell clones to normal mice inhibited the development of liver-stage parasites, underscoring the ability of γδ T-cells to mediate protective immunity in the absence of αβ T-cells [80].

In addition to their role in the liver stage, γδ T-cells have been implicated in various other aspects of malaria immunity. Zaidi et al. (2017) showed that the ablation of γδ T-cells disrupts the accumulation of CD8α+ dendritic cells (DCs), which are essential for priming other T-cell populations [15]. Schofield et al. (2017) further highlighted the suppressive role of TIM-3 expression on γδ T-cells, which impairs their protective immune function during malaria infection [54]. During the blood stage, Farrington et al. (2016) elucidated the role of Vγ9 and Vδ2-expressing γδ T-cells in releasing cytotoxic granules, which support immune responses against infected erythrocytes [72].

Collectively, these findings highlight the multifaceted roles of γδ T-cells in malaria immunity, spanning the liver and blood stages of the parasite’s life cycle. The evidence underscores the importance of these unconventional T-cells in bridging innate and adaptive immunity during malaria infection.

### 4.8. Targeting APCs in T-Cell Activation During Plasmodium Infection

Antigen-presenting cells (APCs), including dendritic cells (DCs) and macrophages, play a crucial role in the pre-activation of T-cells by presenting *Plasmodium* antigens. Co-stimulation with either DCs or macrophages is essential for effective T-cell activation [78]. Toll-like receptors (TLR)-2 and TLR-4 on DC are particularly important for initiating DC maturation and activation, a process driven by IFN-γ, which enhances the immune response to sporozoites [12,13]. Impaired DC maturation can lead to detrimental outcomes, such as the stimulation of Tregs, increased production of the anti-inflammatory cytokine IL-10, and downregulation of co-stimulatory molecules, all contributing to immune insufficiency [18].

### 4.9. The Dual Role of NKT-Cells in T-Cells Activation

Natural Killer T (NKT) cells, traditionally classified as part of the innate immune system, also exhibit characteristics typical of adaptive immune T-cells, highlighting their functional versatility. NKT cells play a crucial role in the immune response against malaria, particularly during the early stages of infection. Prior to the initiation of the adaptive immune response, NKT cells have been shown to exert an antiparasitic effect in the liver, where they actively contribute to the immune defense against liver-stage malaria parasites [17]. The activation of NKT cells is tightly regulated, and studies have demonstrated that the activation of these cells, such as through the use of the glycolipid α-galactosylceramide (α-GalCer), results in a strong, stage-specific inhibition of liver-stage malaria parasites. This protection is IFN-γ dependent, emphasizing the role of NKT cells in inducing a rapid, innate immune response [81]. Furthermore, NKT cells directly influence the adaptive immune response by promoting T-cell activation. One mechanism through which they achieve this is by inducing CD40 expression on conventional dendritic cells (cDC1), which, in turn, co-stimulates CD8+ T-cells, leading to enhanced immune responses [66]. The involvement of NKT cells in this process is further supported by studies showing that their activation can enhance CD8+ T-cell responses and overall anti-malaria immunity when combined with suboptimal doses of irradiated sporozoites or malaria antigen-expressing viruses [82]. This process requires both NKT cell activation and CD1d molecules, as well as the production of IFN-γ, demonstrating the complex interplay between innate and adaptive immune components in malaria. Additionally, NKT cells contribute to the adaptive immune response through granzyme production, which plays a role in the direct cytotoxic response to infected cells, further enhancing their dual role in immune activation and pathogen clearance [30]. These findings underscore the importance of NKT cells not only in the innate immune defense but also in the modulation of adaptive immune responses against malaria and other intracellular pathogens.

### 4.10. The Immunosuppressive Role of Myeloid-Derived Suppressor Cells (MDSCs) in Malaria

Myeloid progenitors are the origin of various immune cells, including monocytes, polymorphonuclear (PMN) cells, and erythrocytes. A subset of these cells, known as myeloid-derived suppressor cells (MDSCs), has the ability to suppress T-cell activity. While regulatory T cells (Tregs) were previously identified as the primary immune suppressors, recent studies highlight the significant immunosuppressive role of MDSCs alongside Tregs [61]. Particularly in malaria, PMN-MDSCs promote the development of Tregs through cytokine-mediated pathways [65]. Understanding the secondary suppression role of PMN-MDSC suggests their potential as a target in malaria immunotherapy to prevent Treg-mediated suppression of T-cell activation.

### 4.11. Methods and Vectors Used in Existing Malarial Immunotherapy

Current strategies for treating infectious diseases, particularly malaria, have increasingly focused on harnessing the human immune system. Unlike traditional drugs that directly target the *Plasmodium* parasite, novel immunotherapy approaches aim to manipulate immune components to enhance immunity [6]. Ongoing studies on malaria have identified several promising methods, including vaccines consisting of the whole parasite or truncated (subunit) vaccines and newer engineered immunizations. The first licensed malaria vaccine, RTS,S, demonstrated moderate efficacy but faced limitations due to the narrow time window in which antibodies can effectively eliminate sporozoites [66]. This is due to the immune defense targeted in RTS,S mainly relies on IgG antibodies during sporozoite challenge, with no evidence of CD8+ T induction, leading to poor cellular immunity [83]. The second licensed vaccine, R21, is still in an early assessment but has shown improved efficacy compared to RTS,S [66].

The literature reviewed in vaccine development trials includes experiments observing both human subjects and inoculated mice with infected red blood cells (Late-stage Parasitized erythrocytes/LP). Several trials also utilized controlled human malaria infection (CHMI) and infection, treatment, and vaccination (ITV) with drug prophylaxis to understand the role of T-cells as a part of adaptive immunity against malaria. The first type of vaccine explored is the whole parasite vaccine, which contains live but attenuated *Plasmodium*, usually in the form of sporozoites. Attenuation methods include irradiation (radiation attenuated sporozoite/RAS), gene modification, or via administration of drugs. Radtke et al. demonstrated that live parasite vaccination favors better CD8α+ DC antigen-presenting activity, suggesting that the parasite’s motility in draining lymph nodes (dLNs) improves antigen capture by antigen-presenting cells (APCs) [16].

Subunit vaccines, in contrast, contain one or more fragments of *Plasmodium* antigen expressed during specific life stages. For example, the circumsporozoite protein (CSP) targets a key surface protein on sporozoites [84], while the multi-epitope thrombospondin-related adhesive protein (ME-TRAP) uses multiple epitopes from various antigens, both primarily targeting CD8+ T-cells during the pre-erythrocytic stage [85]. However, subunit vaccines like CSP face challenges due to antigen polymorphism, where different strains of *Plasmodium* express varied forms of the targeted antigen. This variation can lead to infections caused by strains that are not recognized by the vaccine-induced immune response. Furthermore, clinical trials showed low efficacy, suggesting a vaccine targeting multiple stages is required to induce optimal protective immunity [86]. An exception among vaccines is the Transmission-Blocking Vaccines (TBV) which works by inducing antibodies that target the sexual phase of *Plasmodium*, thus blocking further reproduction within mosquitoes [87].

Subunit vaccines use “vehicles” or vectors to deliver antigens to subjects. In recent decades, pre-erythrocytic subunit vaccines have been developed, including peptide-in-adjuvant, DNA vaccine, and viral vector vaccine. Nevertheless, vaccinations containing peptides in adjuvant subunits are limited to eliciting a strong humoral response and a weak cellular response. In contrast, a modest humoral response but a strong cellular response can be induced by the majority of viral vector subunit malaria vaccines. DNA vaccination is considered simple and stable, but some reports suggest that it may not produce sufficient T-cells or antibodies to effectively eliminate the parasite [88]. One of the challenges with peptide-based vaccines lies in the limited knowledge and technology available to combine human leukocyte antigen (HLA)-restricted epitopes effectively, making broad population coverage difficult to achieve. Ganley et al. proposed mRNA-based vaccines as a promising solution, as they can enable the expression of both whole genes and proteins, potentially offering a wide array of epitopes and increased population coverage [66].

Advanced immunotherapies have also been explored, including the use of monoclonal antibodies (mAbs), a passive immunization approach that involves administering engineered antibodies. These specialized mAbs do not directly target *Plasmodium* but instead function by competitively blocking inhibitory molecules, thereby preventing the suppression of immune responses. Another method is using adoptive cell transfer, which involves isolating immune cells, modifying them, and reinfusing them into the host to strengthen immune response sensitivity [6].

### 4.12. Enhancing and Inhibiting T-Cell to Orchestrate Optimal Immunity Against Malaria

Directing an advanced, boosted immunity against malaria does not imply stimulating all T-cells. Understanding the complexity of T-cells allows insights into whether they should be enhanced or suppressed. As the front-lines during *Plasmodium* immune evasion, CD4+ and CD8+ T-cells are targeted to amplify their magnitude, with potential targets encompassing a wide range of subsets and non-T-cell components that aid defense. Significantly, exceptions such as Treg and MDSC are considered a “brake” of the immune system [61]. Therefore, the proposed immunotherapeutic strategy involves selectively restricting certain cells to optimize the immune response.

In addition to targeting entire cells, certain receptors on T-cells have specialized functions. Studies have identified that stimulatory receptors on T-cells include ICOS and OX40, while inhibitory receptors include CTLA-4, PD-1, LAG-3, TIM-3, and TIGIT. This understanding suggests enhancing co-stimulatory molecules and blocking co-inhibitory pathways. However, consideration is needed to calculate potential adverse effects. Zander et al. demonstrated that simultaneous stimulation of OX40 and blockade of PD-1 can negatively affect parasitic clearance due to excessive production of IFN-γ [60]. A distinction between acute and chronic malaria onset is necessary, as these conditions involve different mechanisms. The co-inhibitory pathway is crucial for limiting the potential for excessive immune responses generated by the host. Thus, blocking this pathway in individuals with acute malaria may lead to immunopathology [56].

## 5. Conclusions

This review provides comprehensive evidence highlighting the critical role of T-cells as a potential target for immunotherapy in malaria. T-cells play a central role in combating malaria by mediating cell-based immunity, fitting the parasite’s intracellular evasion mechanisms, and extensively governing humoral immunity while modulating a broad range of immune components. This review summarizes the contributions of T-cells in malaria, derived from studies on human infections and animal models, both experimental and natural. Understanding the diverse functions of T-cells is essential for devising precise immunotherapy strategies, whether by enhancing existing methods or designing novel approaches.

The evidence collected in this review emphasizes the importance of targeting CD8+ T-cell, which serves as a frontline defense during the liver-stage pre-erythrocytic phase of malaria. This aspect has often been overlooked in previous immunotherapy approaches. Emerging evidence indicates that new immunotherapy vectors, particularly mRNA-based technologies, are leading advancements in enhancing immune responses. However, extensive research is still required to address existing knowledge gaps and to develop improved therapeutic strategies that can contribute to the ultimate goal of malaria eradication.

## Figures and Tables

**Table 1:** Keyword and database used in this study.

Keyword	Database
(“Immunotherapy”) AND (“Immunity, Cellular” OR “Receptors, Antigen, T-Cell” OR “T-Cell Antigen Receptor Specificity” OR “Costimulatory and Inhibitory T-Cell Receptors”) AND (“Malaria” OR “*Plasmodium*”)	Pubmed
	Scopus
	Google Scholar
	Cochrane

**Table 2:** Immunotherapy targets in *Plasmodium* infection based on T-cell activation stages.

No.	T-Cell Activation Stage	Immunotherapy Target	Mechanism of Action	Subject	Study Method	Experimental Result	Reference
1	Pre T-cell-activation	DC	Targeting IFN-γ, which upregulates TLR or directly activates TLR, can enhance DCs to be hyperresponsive to *Plasmodium* and to present antigens more effectively.	*Plasmodium berghei*- and *Plasmodium yoelii 265BY*-infected mice	Experimental RNA- and DNA-vectored vaccines	The effectivity of the immune response to *Plasmodium* infection is enhanced through the induction of dendritic cells by increasing the expression of TLR on dendritic cells, leading to more effective presentation of *Plasmodium* antigens by dendritic cells.	[12,13,14]
2		CD8α+ DC and γδ T-cell	Live parasite vaccines favor better CD8α+ DC antigen-presenting activity due to the parasite’s motility in DLNs. The deficiency of γδT (Vδ2) cells leads to insufficient accumulation of CD8α+ DCs in the liver, impairing the induction of CD8+ T-cell sterile immunity.	*Plasmodium berghei CS^5M^*- and *Plasmodium berghei ANKA*-infected mice	Experimental of CSP vaccine	CD8+ T-cell induction in DLNs is improved by priming CD8a+ DC with live vaccine-derived antigens from *Plasmodium* sporozoites. Whole-sporozoite vaccines confer sterilizing immunity to malaria-naive individuals through mechanisms involving γδ T-cells, which were significantly elevated in protected vaccines. In a mouse model, γδ T-cells were essential for the induction of protective CD8 T-cell responses and sterile immunity, as their absence impaired CD8α+ dendritic cell accumulation in the liver and ablated sterile protection.	[15,16]
3		Natural killer T (NKT) cell	NKT-cell proliferation induces antiparasitic immunity in the liver.	*Plasmodium berghei ANKA*-infected mice	Experimental adjuvants in viral-vectored vaccine and RAS vaccine	The synthetic glycolipid-peptide conjugate vaccine effectively induces long-lived, liver-resident CD8+ T memory cells (T_RM_) as NKT antagonists and provides over 90% sterile protection against *Plasmodium berghei* sporozoite infection for up to 200 days. This highlights its potential as an ideal platform for the control of liver-stage malaria and other hepatotropic infections.	[17]
4		DC	DC maturation critically increases T-cell priming, while impaired maturation stimulates Treg and IL-10, reducing co-stimulatory molecules.	*Plasmodium berghei ANKA*-infected mice	Experimental viral-vectored vaccine and RAS vaccine	Dendritic cells (DCs) treated with cellular extracts from *Plasmodium* berghei-infected erythrocytes (PbX) exhibit impaired maturation and promote the generation of regulatory T-cells.	[18]
5	T-cell activation	CD8+ T-cell	CD8+ T-cell plays a pivotal role in liver sterilizing immunity by providing protection against the pre-erythrocytic stage of infection through the killing of infected hepatocytes (CTLs) and memory of recurrent infections (T_RM_).	*Plasmodium berghei ANKA-*, *Plasmodium chabaudi AS-*, and *Plasmodium yoelii XNL*-infected mice, *Plasmodium falciparum*-infected human adult.	Experimentals of DNA-, cell-, viral-vectored vaccine, VLP vaccine, CSP vaccine, whole parasite vaccine.	In mice and human models, CD8+ T-cell is shown to confer protective qualities in liver stage. Priming CD8+ T-cell is demonstrated by trapping CD8+ T-cell to prime into T_RM_ in liver using prime-and-trap vaccines.	[19,20,21,22,23,24,25,26,27,28,29,30,31,32]
6		T_RM_-like CD8+ T-cell	CD8+ T_RM_-like circulating cells are found to also play role in liver-stage protection.	*Plasmodium falciparum*-immunized human adults	Experimental of viral-vectored vaccine.	The role of T_RM_-like circulating cells in liver contributes to their T_RM_ counterparts; hence, targeting cells with T_RM_ common markers, such as CD69, may improve liver-stage protection by adopting prime-target approach.	[33]
7		CXCR6 receptor on CD8+ T-cell	Upregulation of CXCR6 expression is critical for T_RM_ maintenance, and no correlation with priming or proliferation.	*Plasmodium berghei CS^5M^*-infected mice	Experimental of RAS vaccine.	CXCR6 is crucial for the development and maintenance of protective liver memory CD8(+) T-cells induced by immunization with attenuated malaria sporozoites. While CXCR6-deficient CD8(+) T-cells initially migrate to the liver normally, their numbers significantly decline over time, resulting in a reduced ability to inhibit malaria parasite development in the liver.	[34]
8		CD4+ and CD8+ T-cells	CD8+ T-cell induction is crucial for pre-erythrocytic protection; CD4+ T-cell induction for erythrocytic defense. Elevated function was shown by robust cytokine production, mainly by elevated IFN-γ-producing T-cells.	*Plasmodium yoelii 17X, Plasmodium yoelii 17XNL*-infected mice, *Plasmodium falciparum*-immunized human infants and children, *Plasmodium falciparum*-immunized human adults and pregnant women.	Experimentals of viral-vectored vaccines, whole parasite vaccine; observational of infected and non-infected human.	CD4+ T-cell is critical to induce erythrocytic immunity, whereas CD8+ T executes liver protection immunity. Tailoring vaccines and immunotherapy requires both cells to orchestrate cross-stage immunity against malaria.	[21,35,36,37,38,39,40,41]
9		CD49d receptor on CD4+ T-cell	Th1 to Tfh differentiation is needed for Th/Tfh balance during protection.	*Plasmodium chabaudi*-, *Plasmodium yoelii*-infected mice.	Experimentals of adoptive cell transfer.	CD49d marker is responsible for differentiation from antigen-specific Th1 into Tfh. Stimulating the marker improves immunity by directing the differentiation, leading to elevation of effector cells. Evidence showed better epitope-induced response in mice model. More evidence suggests P2X7 signaling is required to generate balanced Th1/Tfh population with an improved ability to transfer parasite protection to CD4-deficient mice.	[42,43]
10		Tfh CD4+ cell	Tfh is critical in inducing B cell differentiation in germinal center into plasma cells that produce *Plasmodium*-specific antibodies.	*Plasmodium falciparum-*, *Plasmodium chabaudi AS*-, *Plasmodium yoelii*-infected mice, *Plasmodium falciparum*-immunized human adults and children	Experimentals of liposome-, bacteria-vectored vaccines, TBV vaccines, ITV.	T-cell-dependent antibody response requires Tfh expansion to further induce B cell differentiation in germinal center. B-cell clone expansion and transformation into plasma cells enable *Plasmodium*-specific antibodies to contribute to humoral immunity.	[44,45,46,47,48,49]
11		CTLA-4 receptor on regulatory CD4+ T cell	CTLA-4 is an inhibitory receptor suppressing immune response.	*Plasmodium berghei ANKA*-, *Plasmodium falciparum*-infected human adults and children	Experimentals of LP and observational of infected human	Suppression of immunity is proven by the contribution of inhibitory receptor CTLA-1. CTLA-4 receptor works by competing T-cell CD28 receptor binding to CD80/86 on APCs. The upregulation of CTLA-4 in Treg and other CD4+ cells inhibits overall immunity by blocking cell proliferation and antibody production. CTLA-4 is a sign of T-cell anergy and failure to reduce parasitemia.	[50,51,52]
12		TIM-3 receptor on CD4+ T-cell and γδ T-cell	TIM-3 is an inhibitory receptor suppressing immune response.	*Plasmodium berghei ANKA*-infected mice, *Plasmodium falciparum*-infected human adults and children, *Plasmodium vivax*-infected human children	Experimentals of mAb immunization, observational of infected human	TIM-3 negatively regulates cell-mediated immunity. Not only expressed on CD4+ cells, TIM-3 is found on γδ T-cell surface. The upregulation of TIM-3 correlates with T-cell exhaustion. Hence, anti-TIM-3-treatment is executed by blocking TIM-3 signaling with anti-TIM-3-antibody immunization. The result conveys reduced parasite multiplication, resulting in sterile immunity.	[53,54,55]
13		LAG-3 receptor on CD8+ T-cell	LAG-3 is an inhibitory receptor suppressing immune response in chronic malaria. Conversely, LAG-3 is an indicator of elevated function of T-cells in acute malaria.	*Plasmodium berghei ANKA*-, *Plasmodium yoelii*-infected mice	Experimentals of LP.	High levels of PD-1, TIGIT, TIM-3, and CTLA-4 expression were linked with high levels of LAG-3 expression. The highest expression of LAG-3 on antigen-specific CD8+ T-cells appeared to be fully functional during acute malaria, in contrast to what has been reported following chronic antigen exposure. The evidence is seen by the high amount of IFN-γ, GrzB and Perforin.	[56]
14		TIGIT receptor on CD8+ T-cell	TIGIT is an inhibitory receptor suppressing immune response by binding to CD155 or CD112 on APC and outcompeting the co-stimulatory counterpart (CD226). TIGIT signaling facilitates reduced effector function, decreased cytokine production, and impaired cytotoxic activity.	*Plasmodium berghei ANKA*-infected mice	Experimentals of LP.	Suppression of CD8+ T-cell function is by suppression through engagement of co-inhibitory molecules such as TIGIT. T exhaustion occurs due to association of inhibitory molecules. Blockade of co-inhibitory pathway restores T-cell function.	[56,57]
15		PD-1 receptor on CD4+ T-cell	PD-1 is an inhibitory receptor suppressing immune response by binding to PDL-1 on APC, transducing inhibitory signals to T-cells preventing activation, proliferation, and cytokine production pathways.	*Plasmodium berghei ANKA*-, *Plasmodium yoelii 17XNL*-infected mice	Experimentals of peptide and mAb immunization, ITV	PD-1 is a negative immunity regulator component. Upregulation during malaria infection indicates loss of function, reduced proliferation and cytokine production. PD-1 antagonist, LD01 blocks the PD-1:PDL1 interaction. Blockade in Tfh results in elevated antibody production. Blockade in Treg causes reduction in parasitemia.	[44,51,58,59]
16		OX40 receptor on CD4+ and CD8+ T-cells	OX40 is a stimulatory receptor enhancing immune response by binding to OX40L on APC, amplifying intracellular signalling pathways which are crucial for T-cell activation, survival, and differentiation.	*Plasmodium yoelii 17XNL*-infected mice, *Plasmodium falciparum*-, *Plasmodium vivax*-infected human adults	Experimentals of mAb immunization, Observational of infected human	Intervention of engineered monoclonal antibody can be used as a tool to stimulate OX40, a costimulatory signal. Result given is enhanced expansion of CD4+ and CD8+ effector cells and reduced activity of Treg suppression.	[52,59,60]
17		ICOS receptor on CD4+ T-cell	ICOS is a stimulatory receptor enhancing immune response by binding to ICOSL on APC, triggering intracellular signaling cascades leading to enhancement of T-cell proliferation and survival.	*Plasmodium chabaudi AS*-, *Plasmodium yoelii*-infected mice	Experimentals of cell-vectored vaccine	ICOS-ICOSL interaction in Th is critical for Tfh differentiation, proven by effector cell failure in ICOS deficiency. The absence of ICOS depletes the ability to induce Tfh, weakening humoral immunity. Vaccine enhancement in ICOS signaling pathway increases immunity in blood-stage protection.	[48,56]
18		Regulatory CD4+ T-cell	Treg suppresses immune response by binding to inhibitory receptors and producing anti-inflammatory cytokines (IL-10)	*Plasmodium berghei ANKA*-, *Plasmodium yoelii 17XNL*-infected mice and tumor-bearing mice, *Plasmodium falciparum*-, *Plasmodium vivax*-infected human adults	Experimentals of peptide and mAb immunization and observational of infected human	Treg executes immunosuppressive function against malaria, depleting immune response in CSP malaria vaccine, especially CD8+ T. Blockade of inhibitory molecules expressed on Treg is an approach to prevent Treg in dampening immune response.	[18,50,52,58,61,62]
19		CD4+ T-cell	Balance of Th17/Treg is needed for immune homeostasis. The cytokines assiciated are TGF-β, IL-6, and IL-10.	*Plasmodium berghei ANKA-*, *Plasmodium yoelii 17XNL*-infected mice	Experimentals of LP.	Th17 and T regulatory (Treg) cells have opposing impacts on host immune responses, thus highlighting the balance importance. TGF-β and IL-10 are key regulators of Treg, whereas IL-6 and TGF-β coordinated by STAT-3 supports Th17 production. Cytokines association evidence is established by anti-TGF-β and anti-IL-6 treatments.	[62,63]
20		Th2 CD4+ cell	Plasticity of Th2 enables conversion into Th2, producing IFN-γ in helminth co-infection. Evident antagonist relation of Th1/Th2.	*Heligmosomoides polygyrus* and *Plasmodium chabaudi* co-infected-mice	Experimentals of LP.	Helminth and *Plasmodium* induce very opposing and non-interchangeable immune responses. Prominent Th2 and IgE activity is required during helminth defence but is reduced due to *Plasmodium* infection by TCR, IL-12, and IFN-γ turning Th2 into IFNγ-secreting cells, compromising anti-helminth protection yet increasing anti-*Plasmodium* immunity.	[64]
21		Myeloid-derived suppressor cells (MDSC)	MDSC is shown to have suppressive effect by blocking T-cell response via supporting Treg	*Plasmodium yoelli 17XNL*-infected tumor-bearing mice, *Plasmodium falciparum*-immunized human adults.	Experimentals of LP and CHMI.	PMN-MDSC supports the development of Treg and performs cytokine pathways to blockade T-cell response, inhibiting optimal immune response. Blockade therapy of both Treg and MDSC results in enhanced T-cell immunity.	[61,65]
22		CD40 receptor on DC	NKT-cell induces co-stimulation of Tmem by stimulating CD40 on DC.	*Plasmodium berghei ANKA*-infected mice	Experimental mRNA-vectored vaccine	Vaccination with glycolipid–peptide method allows NKT recruitment. NKT signals rapid priming of T_RM_. NKT antagonist enhances costimulation on DC and CD8+ T-cells by targeting CD40. The result reached 80% of T_RM_ in proportion to T_EM_ and T_CM_ on liver in mice model.	[66]
23	Post T-cell activation	CD4+, CD8+ T-cell and γδ T-cell	Teff conversion ability into Tmem decreases after reaching late phase Teff. Tmem is important for re-infection protection.	*Plasmodium chabaudi AS*-infected mice, *Plasmodium falciparum*-immunized human adults.	Experimentals of LP.	Re-infection immune response is enhanced by inducing more Tmem. Conversely, Teff is important in current protection. FAS pathway induces 68.4% of genes of Tmem in proportion to Teff. Chemically attenuated *Plasmodium* vaccine increases Teff with significant increase in cells secreting IFN-γ alone and triple cytokine-secreting cells (IFN-γ, TNF, and IL-2), albeit at lower frequency.	[67,68,69]
24		AT_1_R receptor in CD8+ T-cell	Signaling of AT_1_R results in increased cytokine production, regulates effector and memory phase, and decreases T-cell exhaustion.	*Plasmodium berghei CS^5M^*-infected mice	Experimental viral-vectored vaccine	Host malarial immune response is enhanced by stimulating AT_1_R receptor on CD8+ T-cells. Mice infected with blood-stage PbA are protected against lethal malaria by parasite-specific CD8+ T lymphocytes that do not express AT1R. Survival in AT1R deficient mice only lasted for 7 days, whereas wild-type mice with AT1r preserved survival until day 26.	[70]
25		NKT-cell, monocyte, CD4+ T-cell and γδ T-cell	Acquired innate immune in BCG vaccination enhances adaptive immune response towards malaria, shown by increased granzyme B activity.	*Plasmodium falciparum*-immunized BCG-immunized human adults.	Experimental of CHMI	Induced innate response due to BCG vaccination in malaria-infected volunteers showed prior increase of IFN-γ, granzyme B, inflammatory C-reactive protein (CRP), leading to strong lymphocyte and monocyte activation. The robust response correlates with lower parasitemia.	[71]
26		γδ T-cell	Vγ9 and Vδ2 chain controls blood stage by secreting cytotoxic granules in early childhood malaria.	*Plasmodium falciparum*-immunized human children	Experimental of CHMI	Randomized controlled trials of age groups have shown decreasing levels of Vγ9Vδ2 T and declining percentages of IFN-γ/TNF-α^+^ and CD107a^+^ Vδ2 cells with increasing age. The data proves the decreasing ability of Vδ2 T-cells to secrete proinflammatory cytokines and degranulate in response to *P. falciparum* antigen based on observations, suggesting that the functional capacity of these cells diminishes with age.	[72]

**Abbreviations**: APC: Antigen-Presenting Cell; AT1R: Angiotensin II Type 1 Receptor; BCG: Bacillus Calmette-Guérin; CHMI: Controlled Human Malaria Infection; CRP: C-Reactive Protein; CSP: Circumsporozoite Protein; CXCR6: C-X-C Motif Chemokine Receptor 6; DC: Dendritic Cell; DLNs: Draining Lymph Nodes; GrzB: Granzyme B; IFN-γ: Interferon Gamma; IL-10: Interleukin 10; IL-12: Interleukin 12; IL-2: Interleukin 2; IL-6: Interleukin 6; ITV: Infection, Treatment, and Vaccination; LP: Late-stage Parasitized erythrocytes; mAb: Monoclonal Antibody; MDSC: Myeloid-Derived Suppressor Cells; NKT: Natural Killer T-cell; OX40: Tumor Necrosis Factor Receptor Superfamily, Member 4; PD-1: Programmed Cell Death Protein 1; PDL-1: Programmed Death Ligand 1; RAS: Radiation-Attenuated Sporozoites; STAT-3: Signal Transducer and Activator of Transcription 3; TBV: Transmission-blocking Vaccine; T_CM_: Central Memory T-cell; Teff: Effector T-cell; T_EM_: Effector Memory T-cell; Th: T-helper Cell; TLR: Toll-like Receptor; TIGIT: T-cell Immunoreceptor with Ig and ITIM Domains; TIM-3: T-cell Immunoglobulin and Mucin-Domain Containing-3; Tmem: Memory T-cell; T_RM_: Tissue-Resident Memory T-cell; Treg: Regulatory T-cell.

## Data Availability

Not applicable.
